# Determination of Mercury Content in Surface Waters Using an Environmentally Non-Toxic Terminating Electrolyte

**DOI:** 10.1007/s00128-020-02992-w

**Published:** 2020-09-21

**Authors:** Joanna Jabłońska, Mariusz Kluska

**Affiliations:** Faculty of Exact and Natural Sciences, Siedlce University of Natural Sciences and Humanities, 54 3-Maja St, 08-110 Siedlce, Poland

**Keywords:** Surface water, Mercury, Isotachophoresis, Silanates

## Abstract

The paper presents results of the research on the dynamics of changes in the concentration of mercury in surface waters. The importance of mercury as an environmental pollutant results from specific properties of this metal, many sources of contamination, volatility, mobility, stability and high toxicity of its specific chemical forms. Samples of surface water collected from three rivers: the Bug, the Liwiec and the Muchawka were analysed. The Muchawka River flows into the Liwiec River, which in turn is a tributary of the Bug River. The technique of isotachophoresis was employed, using a solution of a biodegradable and environmentally non-toxic derivative of electrostatically stabilised silanates as the terminating electrolyte. The highest average mercury concentration of 0.89 μg/dm^3^ was determined in water samples collected from the Bug River in January, whereas the lowest concentration of 0.42 μg/dm^3^ was recorded in water collected from the Muchawka River in September.

Mercury occurs in the natural environment. If the content is small and mercury constitutes only a component of minerals, it does not pose a significant threat to living organisms (Siudek et al. 2016a, b; Borzyszkowski and Gworek 2016; Kowalski et al. 2007; Kluska et al. 2007). However, the problem is human activity involving the release of large amounts of mercury into the environment, which may remain there for many years. The main problem is mercury contained in water and sediment, as it occurs there in a highly toxic form and can be taken up by animals, thus entering the human food chain. The World Health Organisation has identified 10 chemical substances that pose a serious threat to health, four of which are heavy metals: mercury, lead, cadmium and arsenic (Michalski et al. 2018; 2019; Kluska et al. 2014; Siudek et al. 2016a, b).

Human activity to date has led to the release of hundreds of thousands of tonnes of mercury into the environment. It is estimated that the level of mercury in the atmosphere is now five times higher than the natural level and the concentration of mercury in the oceans is about twice as high as the natural level. Therefore, much attention is being paid to the spread of mercury in all environmental compartments. This chemical element has been added to the list of priority pollutants by various organizations and programmes aimed at reducing the emission of heavy metals to the environment (Gworek et al. 2017; Nawała et al. 2016).

Despite significant emission reductions, mercury is still widespread in the environment, in all its compartments and in the entire trophic chains. Mercury enters into the animal and seabird body in the most toxic organic form, i.e. methylmercury, mainly through the digestive tract. Once in the body, it turns into less toxic forms and accumulates in internal organs. The problem, however, consists in the fact that it is not biodegradable and forms many toxic compounds, both organic and inorganic. Moreover, it also enters the human body in significant quantities (Giacomino et al. 2017; Kwaansa-Ansah et al. 2016; Kluska et al. 2015; Jabłońska and Kluska 2019).

Contamination with mercury and its toxic forms may cause various adverse health effects for all living organisms. They can occur both immediately after exposure or after certain time, and the latter effects are referred to as “long-term toxic effects”. Mercury is considered a persistent environmental pollutant, because it is not transformed into harmless forms and is classified as a global pollutant affecting the environment, including the human body (Hagemann et al. 2014; Xia et al. 2010; Stoichev et al. 2006).

The distribution of mercury and its forms in organs depends on the duration of exposure and the type of compound absorbed by a given organism. The mechanism of toxic effects of mercury and its compounds indicates that the cell membrane is the first place attacked by mercury. Sulfhydryl groups in cell membranes show a high degree of affinity with mercury and its compounds. Almost all proteins contain sulfhydryl groups and readily react with metals, including mercury. As a result, mercury compounds may interfere with most enzymatic reactions (Siudek et al. 2016a, b; Borzyszkowski and Gworek 2016; Małkiewicz et al 2015).

Mercury accumulates in surface water due to natural erosion and industrial waste. It also often reaches the oceans, hence fish are among the most common sources of exposure to mercury. The risk associated with the consumption of certain fish is particularly high for pregnant women, as numerous studies indicate a correlation between mercury and neurodevelopmental disorders in the foetus (Siudek et al. 2016a, b; Clarkson and Magos 2006).

Mercury and its compounds are potentially involved in four main biochemical processes in cells, leading to genotoxicity: formation of free radicals and oxidative stress, impact through microtubules, influence on the DNA repair mechanisms, direct impact on the DNA (Gworek et al. 2017).

Despite previous reductions in the use and emission of mercury in Europe and North America, its level in the environment is likely to remain high for a long time. This is due to the long-term persistence of mercury in the environment and increased emission of mercury in other regions of the world. As mercury can travel over long distances, it is estimated that almost half of the mercury deposited in Europe comes from outside the continent. All of this creates an obligation for analysts to monitor the level of concentrations of individual forms of mercury in the environment in the most comprehensive way possible. The objective of the study was to determine the concentration of mercury in surface waters of three rivers using an environmentally non-toxic and biodegradable terminating electrolyte. In the study, the isotachophoresis technique was used as a green alternative to ion chromatography and other classical measurement methods (Kluska et al. 2009a, b; Jabłońska et al. 2018; Prukała et al. 2008).

## Materials and Methods

In order to determine the dynamics of changes in the content of mercury, a total of 15 samples of surface water were collected from three rivers—five samples from each river—at four time intervals, i.e. in January, April, June and August 2019. The three rivers involved in the study are: the Muchawka River (samples were collected in the town of Siedlce), the Liwiec River (samples were collected in the town of Węgrów) and the Bug River (samples were collected in the town of Wyszków). The Muchawka River is 32.1 km long and is a left-bank tributary of the Liwiec River, while the length of the Liwiec River is 126.3 km and it is the longest tributary of the Bug River. The catchment area of the Liwiec River is 2780 km^2^. The chemical status of water in the Muchawka and Liwiec rivers is described as moderate. In the Liwiec River, mainly the annual average and maximum concentrations of indeno[1,2,3-c,d]pyrene and benzo[g,h,i]perylene were exceeded (Borzyszkowski and Gworek 2016).

**Fig. 1:**
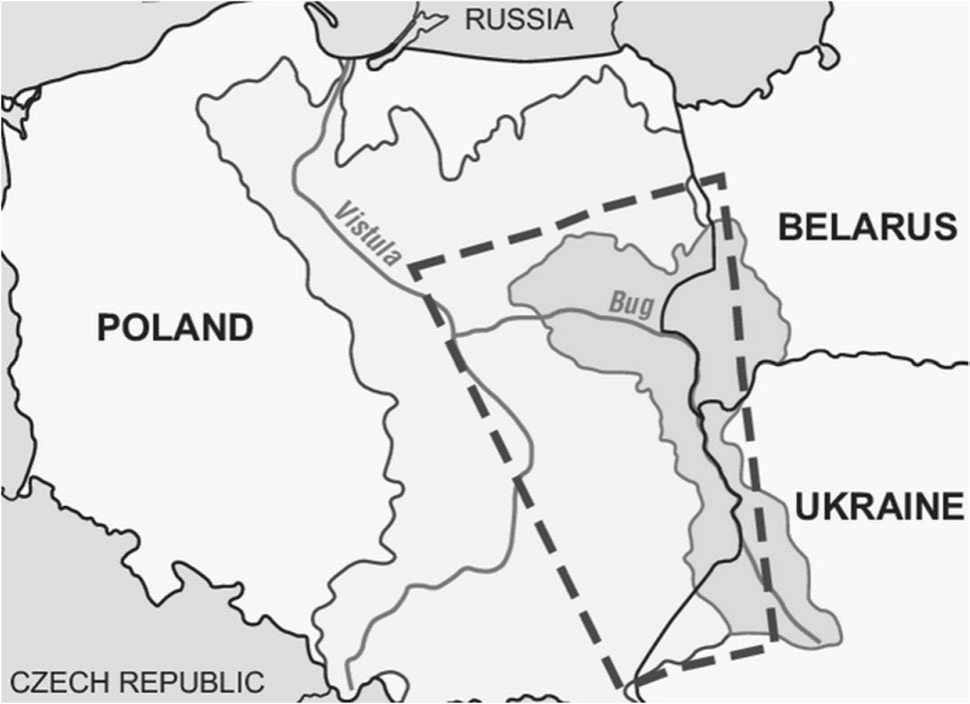
Surface water sampling area

The Bug River (Fig. 1), on the other hand, is a border river with Ukraine along a long section and receives large amounts of wastewater. In the Mazovia Province, the largest source of pollution for this river is the town of Wyszków, which discharges about 3000 m^3^ of wastewater per day from a sewage treatment plant with improved nutrient removal. In addition, large loads of pollutants are discharged to the Bug River from the Toczna and Cetynia rivers (sewage from the Sokołów Podlaski WWTP). A typical contamination of the Bug River are total suspended solids. The pH of all water samples was slightly alkaline during the sampling periods and the mean values were: pH 7.23 for the Muchawka River, pH 7.58 for the Liwiec River and pH 8.31 for the Bug River.

Prior to the analysis, a standard solution was prepared, which was potassium tetraiodomercurate—K_2_[HgI_4_] dissolved in deionised water at a concentration of 0.1 mg Hg/cm^3^. Then the working solution was prepared by diluting the standard solution 100 times and 1 cm^3^ of K_2_[HgI_4_] solution was pipetted into a 100 cm^3^ volumetric flask and made up to the calibration mark with deionised water and well mixed. The following volumes of the working solution were then successively pipetted into 5 cm^3^ volumetric flasks: 0.0; 0.2; 0.4; 0.6; 0.8; 1.0 cm^3^, topped up with deionised water and mixed together. The prepared standard solutions were analysed and the analytic curve was determined.

The next stage of the analysis was to convert the mercury contained in the analysed surface water samples into a complex of potassium tetraiodomercurate. To this end, potassium iodide solution was slowly added to each water sample of 0.5 dm^3^ filtered through a filter paper with a 5 µm pore diameter, stirring carefully to ensure that the mercury contained in the samples was complexed. As described in the literature (Kluska et al. 2007), a small amount of HgI_2_ precipitate was formed initially, which with an excess of potassium iodide dissolved to form a colourless solution of K_2_[HgI_4_]. The thus prepared solutions were introduced into the isotachophoresis apparatus.

**Fig. 2:**
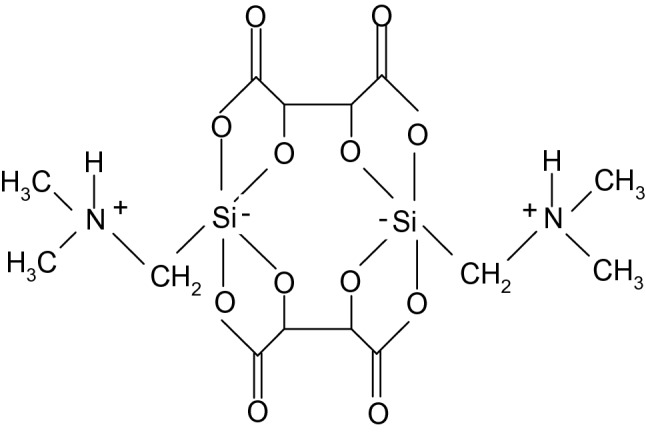
Structure of a compound used in terminating electrolyte 4,4′-bis{1-[(*N,N*-dimethyl)aminiomethyl]spirobi(1-sila-2,5-dioxacyclopentan-3-on)at}

Only analytical grade reagents were used in the analysis. In accordance with the literature, the leading electrolyte (LE-1) was a mixture of equal volumes of solutions: 8·10^–3^ mol/dm^3^ NaCl (POCH Gliwice), 3·10^–3^ mol/dm^3^ BIS–TRIS propane (Aldrich), 1.5·10^–3^ mol/dm^3^ β-alanine (Aldrich) and 0.1% hydroxyethyl cellulose (Aldrich). The leading electrolyte (LE-2) was prepared by mixing equal volumes of solutions: 2·10^–3^ mol/dm^3^ NaCl, 1.5·10^–3^ mol/dm^3^ β-alanine and 0.1% hydroxyethyl cellulose (Merck) for column coating. Apart from that, deionised water (Merck) and potassium tetraiodomercurate (POCH Gliwice) were used. As the terminating electrolyte (TE), 5·10^–3^ mol/dm^3^ solution of 4,4′-bis{1-[(*N,N*-dimethyl)aminomethyl]spirobi(1-sila-2,5-dioxacyclopentane-3-on)at} was used (Fig. 2) (Kluska 2008; Jabłońska et al. 2020a).

A standard isotachophoresis and capillary electrophoresis analyser (EA 202 M, Villa Labeco, Slovakia) was used in the study, along with a personal computer with the ITPPro 32 software as a control unit. The analyser consists of: an injection valve, a container with the terminating electrolyte, a preseparation column, a branching block, a counter electrode container, a preseparation column, an analytical column, a UV detector and two conductivity detectors with a measuring range of 30 kΩ to 20 MΩ.

## Results and Discussion

Mercury occurs in surface waters mainly in the ionic form. The solubility of metallic mercury in water is very poor, with a maximum of 0.056 g/dm^3^ of pure water, and it is even lower in ocean water. When determining the mercury contained in surface water samples, special attention should be paid to the possible presence of ammonia. This is due to the fact that, according to the mercury determination methodology described above, the complex of potassium tetraiodomercurate very easily reacts with ammonia, and then part of the determined ions [HgJ_4_]^2−^ would be precipitated with ammonia and the results of the analysis would be significantly incorrect. When [HgJ_4_]^2−^ ions react with ammonia, a solution turns yellow. At higher concentrations of ammonia in water samples, brown precipitate would be formed as evidence of the presence of ammonium ions.

The content of ammonia in surface waters varies considerably during the year. The concentration values decrease in summer, when it is assimilated by plant organisms and when nitrification processes are intense. In the winter season, however, both vegetation and nitrification are inhibited, so that the content of ammonia shows an increasing trend. Based on the content of individual ionic forms of nitrogen present in water [ammonium ion, nitrate(V) and nitrate(III)] and the analysis of their variability over time, it is possible to make an approximate assessment of the time and possible origin of pollution.

The high level of the ammonium ion in the absence of nitrate(III) ions indicates recent surface water pollution, where municipal wastewater is predominant. The simultaneous presence of ammonium and nitrate(III) ions indicates that water has been contaminated in the relatively recent past. On the other hand, the absence of ammonium and nitrate(III) ions in water along with elevated concentrations of nitrate(V) ions suggests that contamination has occurred quite a long time ago. Moreover, it indicates that the time elapsed since the pollutants penetrated into the water was long enough to allow for the oxidation of ammonium ions. This configuration also indicates the likely contamination of water as a result of run-off from agricultural areas.

Therefore, the method of mercury analysis consisting in the formation of potassium tetraiodomercurate makes it possible to determine both the content of ammonia as well as when and where this contamination originated. Isotachophoresis can also be employed to analyse anions, without changing the terminating electrolyte using a derivative of electrostatically stabilised silanates (Jabłońska et al. 2020b).

**Fig. 3:**
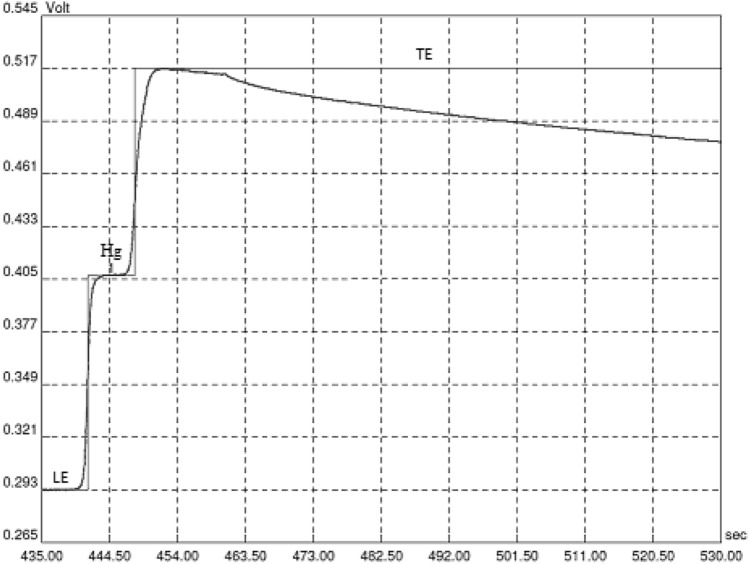
One of the isotachophoregrams obtained during the analysis of standard mercury solutions (for the conditions of the analysis see Table 1b)

**Fig. 4:**
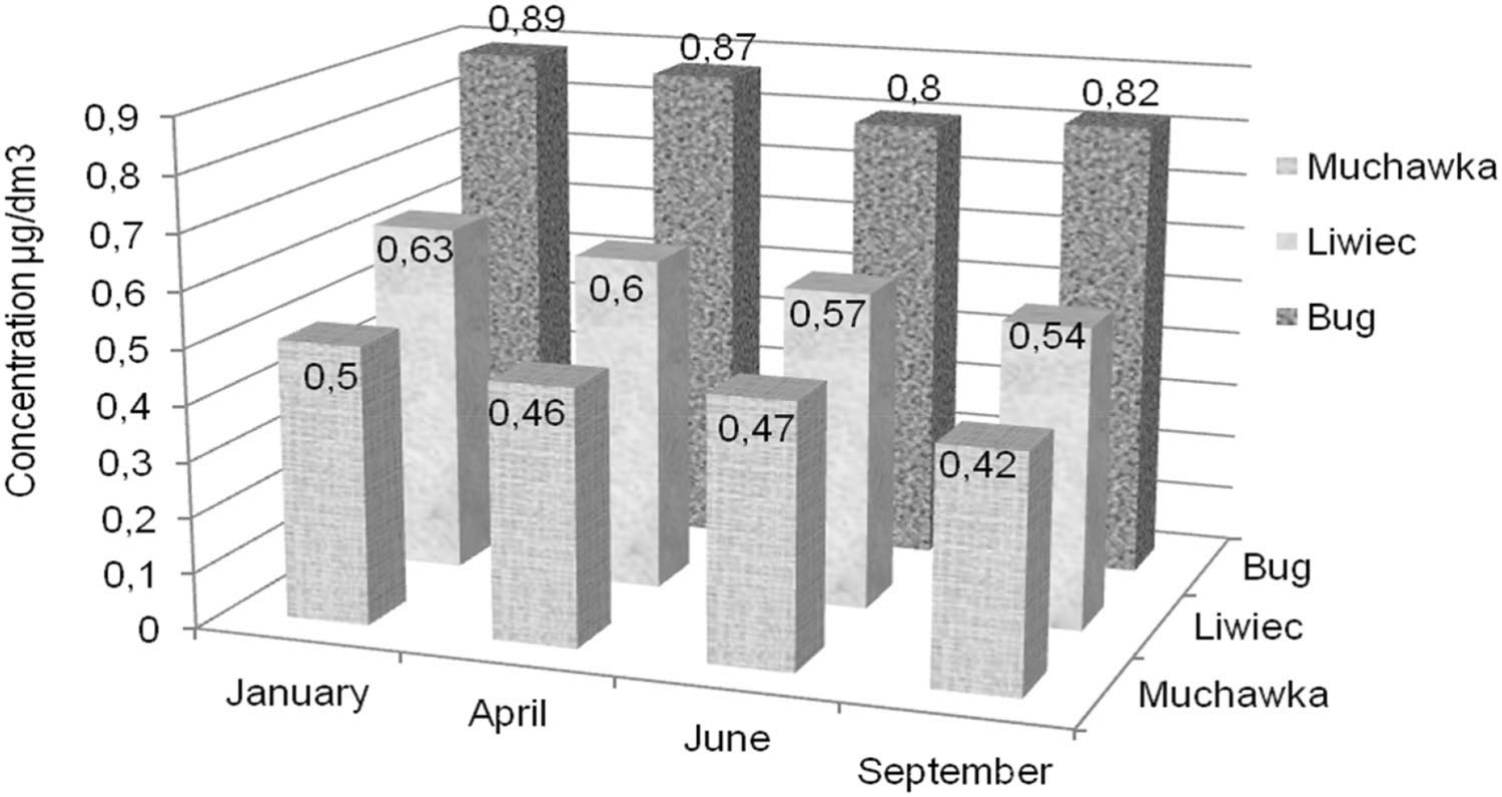
Dynamics of changes in the content mercury over time in surface waters of each surveyed river

The use of isotachophoresis for the determination of ionic forms of various substances from aqueous solutions does not require condensation of a solution, as this process occurs automatically during the conducted analysis. The data characterising the applied analytical method and the obtained results are presented in Tables 1, 2 and 3 and Figs. 3 and 4.

**Table 1:** Specific common conditions of the method used for the calibration curve and the analysis of mercury in the collected surface water samples

(a)	
Parameters of the method	
Sample rate [smp/s]	50
Polarity	+ cations
High voltage limit [V]	12,000

(b)				
Step	Time [s]	Intensity [µA]	Comp [10 mV]	Conductometric detector
1	100	200	0	–
2	260	150	50	–
3	170	250	0	X

**Table 2:** Parameters taken under consideration during the validation process

Parameter	In conversion to Hg
Recovery	94 ± 5%
Precision	4.8%
Limit of detection	0.03 µg/dm^3^
Limit of quantification	0.10 µg/dm^3^
Linearity	0.1‒10.0 µg/dm^3^

**Table 3:** Average mercury content in the tested water samples (n = 5)

Water sample from the River:	January [μg/dm^3^] ± RSD [%]	April [μg/dm^3^] ± RSD [%]	June [μg/dm^3^] ± RSD [%]	September [μg/dm^3^] ± RSD [%]
Muchawka	0.50 ± 3.8	0.46 ± 2.9	0.47 ± 4.2	0.42 ± 4.5
Liwiec	0.63 ± 3.3	0.60 ± 3.7	0.57 ± 3.9	0.54 ± 3.4
Bug	0.89 ± 3.4	0.87 ± 4.3	0.80 ± 4.4	0.82 ± 4.0

*RSD* relative standard deviation

Other methods are often used to analyse mercury from aqueous solutions, including, inter alia, the extraction and colorimetric method, flameless atomic absorption spectrophotometry combined with the cold vapour method (CV-AAS) and the indirect dithizone method with copper carbamate. The methods of mercury determination have been compared a number of times (Giacomino et al. 2017; Kwaansa-Ansah et al. 2016; Xia et al. 2010; Domanico et al. 2017). In this study, however, the analysis was limited to isotachophoresis following the previously developed methodology (Kluska et al. 2014). The optimal parameters for qualitative and quantitative analysis are presented in Table 1.

The method applied has been validated according to the literature (Ermer and Agut 2014). The following parameters were determined: recovery, precision, linearity, the limit of detection and the limit of quantification. The prepared standard solutions were used to characterise the analytical method employed and to create the calibration (standard) curve. The results obtained during the validation process of the applied analytical method are collated in Table 2. One of the isotachophoregrams obtained during the analysis of standard solutions for the purpose of drawing up the standard curve is presented in Fig. 3. When analysing the results obtained, it can be concluded that this method is distinguished by good validation parameters, such as high precision and a wide range of linearity. The detection limit of the applied method is 0.03 µg/dm^3^ and the quantification limit is 0.10 µg/dm^3^. Therefore, the method can be used for the determination of mercury in the analysed surface waters.

Determination of mercury so as to obtain reliable results is not an easy task. This process is primarily determined by the proper stage of sampling, storage and preparation of a sample as well as the application of a relevant analytical technique. These aspects are particularly important when analysed samples do not originate from places exposed to strong anthropopressure, for example surface or ground waters unpolluted by mercury. These difficulties may be even greater in the case of speciation analysis, as additional activities to ensure the stability of individual chemical forms must be considered (Siudek et al. 2016a, b; Michalski et al. 2019).

Pursuant to the Ordinance of the Minister of Maritime Economy and Inland Navigation of 29 November 2019 on quality criteria for surface waters used as a source of drinking water, the permitted mercury content is 1 µg/dm^3^. The results obtained for the surface water samples are below the limit values for mercury (Table 3). The highest average content of mercury was found in samples collected from the Bug River, regardless of the sampling date. And thus, the average content in samples collected in January was at the level of 0.89 μg/dm^3^, in April—0.87 μg/dm^3^, in June—0.80 μg/dm^3^ and in September—0.82 μg/dm^3^. On the other hand, the lowest mercury content in the studied period was recorded in the Muchawka River, i.e. 0.50 μg/dm^3^ in samples collected in January, 0.46 μg/dm^3^—in April, 0.47 μg/dm^3^—in June and 0.42 μg/dm^3^—in September.

The obtained data (Table 3) show that the content of mercury in basically all surface water samples has slightly decreased over time (Fig. 4). On the other hand, a slight increase in the dynamics of mercury content can be observed along the surveyed section of surface waters, i.e. the Muchawka River, the Liwiec River and the Bug River. The highest mercury content was recorded in January and the lowest in September. This relationship applies to samples collected from all three rivers. Very similar average values of mercury content were recorded in waters collected from the Liwiec River, regardless of the month of sampling. These values ranged from 0.63 μg/dm^3^ in January to 0.54 μg/dm^3^ in September. All the obtained results using isotachophoresis showed low values of standard deviation, i.e. below 5%. Low standard deviation values indicate high precision of the analytical method used. Slightly lower values of concentrations were obtained by other authors of miscellaneous studies conducted in their respective countries, both Europe and other parts of the world (Giacomino et al. 2017; Kwaansa-Ansah et al. 2016).

To date, surface waters have been analysed many times, but the content of mercury was not usually included. The developed method will provide a basis for extending the analytics to further mercury analytics and will enable a wider range of research of these waters in the coming years. In subsequent studies, this will allow for a better statistical evaluation and a more accurate comparison of seasonal changes.

To sum up, it is worth emphasising that water degradation prevention should be comprehensive and cover all spheres of human activity. The process of preventing water degradation is usually very expensive and, therefore, should be supported by various prohibitions and orders of water law and regulations of the Ministry of Maritime Economy and Inland Navigation.

## Conclusions

In all samples collected from surface waters for laboratory analysis, the content of mercury ranged from 0.42 µg/dm^3^ in September in the Muchawka River to 0.89 µg/dm^3^ in January in the Bug River. The content of mercury was successfully analysed by isotachophoresis, using an environmentally non-toxic terminating electrolyte belonging to electrostatically stabilised silanates. The dynamics of changes in the mercury content decreased with the passage of time, but slightly increased along the surveyed section of the surface waters. The low content of mercury in the surface water samples determined in this study indicates an insignificant impact of human activity.
